# Recurrent Urinary Tract Infection in Adult Patients, Risk Factors, and Efficacy of Low Dose Prophylactic Antibiotics Therapy

**DOI:** 10.1007/s44197-023-00105-4

**Published:** 2023-06-05

**Authors:** Hala Alghoraibi, Aisha Asidan, Raneem Aljawaied, Raghad Almukhayzim, Aljoharah Alsaydan, Elaf Alamer, Waleed Baharoon, Emad Masuadi, Abeer Al Shukairi, Laila Layqah, Salim Baharoon

**Affiliations:** 1 College of Medicine, King Saud Ibn Abdulaziz University for Health and Sciences, Riyadh, Saudi Arabia; 2 King Abdullah International Medical Research Center, Riyadh, Saudi Arabia; 3 Department of Intensive Care, King Abdulaziz Medical City, Riyadh, Saudi Arabia; 4 King Abdullah International Medical Research Center, Riyadh, Saudi Arabia; 5 King Saud Bin Abdul-Aziz University for Health Sciences, Riyadh, Saudi Arabia; 6 Ministry of National Guard-Health Affairs, Riyadh, Saudi Arabia; 7 Department of Medicine, King Faisal Specialist Hospital and Research Center, Jeddah, Kingdom of Saudi Arabia; 8 College of Medicine, Al Faisal University, Riyadh, Kingdom of Saudi Arabia; 9 College of Medicine, United Arab Emirates University, Al Ain, United Arab Emirates; 10 College of Dentistry, King Saud Bin Abdulaziz University for Health Sciences, Riyadh, Saudi Arabia

**Keywords:** Urinary tract infection, Recurrent urinary tract infection, Antibiotic prophylaxis, Risk factors, TMP-SMX, Nitrofurantoin

## Abstract

**Background:**

Recurrent urinary tract infection (UTI) occurs in sizable percentages of patients after a single episode and is a frequent cause of primary healthcare visits and hospital admissions, accounting for up to one quarter of emergency department visits. We aim to describe the pattern of continuous antibiotic prophylaxis prescription for recurrent urinary tract infections, in what group of adult patients they are prescribed and their efficacy.

**Methods:**

A retrospective chart review of all adult patients diagnosed with single and recurrent symptomatic urinary tract infection in the period of January 2016 to December 2018.

**Results:**

A total of 250 patients with a single UTI episode and 227 patients with recurrent UTI episodes were included. Risk factors for recurrent UTI included diabetes mellitus, chronic renal disease, and use of immunosuppressive drugs, renal transplant, any form of urinary tract catheterization, immobilization and neurogenic bladder. *E. coli* infections were the most prevalent organism in patients with UTI episodes. Prophylactic antibiotics were given to 55% of patients with UTIs, Nitrofurantoin, Bactrim or amoxicillin clavulanic acid. Post renal transplant is the most frequent reason to prophylaxis antibiotics (44%). Bactrim was more prescribed in younger patients (*P* < *0.001*), in post-renal transplantation (*P* < *0.001*) and after urological procedures (*P* < 0.001), while Nitrofurantoin was more prescribed in immobilized patients (*P* = *0.002*) and in patients with neurogenic bladder (*P* < 0.001). Patients who received continuous prophylactic antibiotics experienced significantly less episodes of urinary tract infections *(P* < *0.001*), emergency room visits and hospital admissions due to urinary tract infections (*P* < *0.001*).

**Conclusion:**

Despite being effective in reducing recurrent urinary tract infection rate, emergency room visits and hospital admissions due to UTI, continuous antibiotic prophylaxis was only used in 55% of patients with recurrent infections. Trimethoprim/sulfamethoxazole was the most frequently used prophylactic antibiotic. Urology and gynecological referral were infrequently requested as part of the evaluation process for patients with recurrent UTI. There was a lack of use of other interventions such as topical estrogen in postmenopausal women and documentation of education on non-pharmacological methods to decrease urinary tract infections.

## Introduction

Urinary tract is a leading site of infections in all ages and genders. It is prevalent as a community and healthcare related infections and affects both immunocompetent and immunocompromised hosts [1–4]. The highest prevalence is among women, with at least one episode of urinary tract infection (UTI) affecting up to 50% of females in their lifetime [5]. Urinary tract infections are frequent causes of primary healthcare visits and hospital admissions, accounting for up to one quarter of emergency department visits especially in certain high-risk groups [6–9]. The overall prevalence of uncomplicated urinary tract infection in USA is between 8 and 11% [7, 8, 10] while that of women over age of 65 is 20%. In Saudi Arabia, prevalence of urinary tract infection ranges from 9.8 to 24% in some publications [11–13] but no national estimation is available.

Recurrent urinary tract infection, defined as the occurrence of three episodes of UTIs in 12 months or two episodes in 6 months incidence may be as high as 44% [6, 9, 10, 14]. After a first episode of an uncomplicated urinary tract infection, at least one second episode will occur in 27% of women in the next 6–12 months [5]. Risk factors for recurrences includes history of childhood UTI [odds ratio (OR = 6.8)] back-to-front douching/wiping after bowel movement (OR = 2.6), younger age at first intercourse (OR = 6.3), increased frequency of sexual intercourse (OR = 4.8), obstructed urinary flow (OR = 1.9), and genital prolapse (OR = 3.4) and lower Vitamin D level [15, 16]

Prevention of recurrent UTIs includes continuous and postcoital antimicrobial prophylaxis and the use of topical estrogen in postmenopausal women [17–21]. Antimicrobial regimens commonly used for continuous antimicrobial prophylaxis include Bactrim, Fosfomycin and Nitrofurantoin. Less frequently used medications include first generation cephalosporin Cephalexin and Amoxicillin/clavulanic acid.

The prescription patterns of continuous antimicrobial prophylaxis in children and adults are not well described in Saudi patients. Barry et al. reported on status of long-term antibiotic prophylaxis for urinary tract infections in children including 34 RCTs, 9 systematic reviews, and 3 guidelines with no reference to any literature from Saudi Arabia [20]. We seek to describe the pattern of antibiotic prescription, type, duration, prescribers and in what group of patients, in adults’ Saudi patients with recurrent urinary tract infection and to assess how effective the treatment were compared to those who were not given continuous prophylaxis.

## Method

The study was conducted at King Abdulaziz Medical City, National Guard-Health affairs (NGHA), Riyadh. This is one the major tertiary care medical cities in Saudi Arabia where it is estimated that more than a 100,000 patient visits occur annually in Outpatient department (OPD).

Electronic heath record (BESTCare system) review was retrospectively performed searching all patients age 18 years or older with at least one episode of symptomatic urinary tract infection from January 2016 to December 2018. Only outpatient visits were included. We excluded patients who are pregnant at the time of UTI diagnosis. All patients should have a minimum 12 months of follow-up after the first diagnosis of UT.

Patients were then grouped into those with single urinary tract infection during the follow-up of one year from initial episode and those with recurrent infections. Recurrent urinary tract infection is defined as either ≥ 3 symptomatic episodes with positive urinary cultures per year or ≥ 2 symptomatic episodes with positive urinary cultures in the last 6 months. Patients who were started on continuous prophylactic antibiotics were identified from the group with urinary tract infection. In those patients, a minimum follow-up of 12 months post completion of the antibiotic regimens was required.

The following data were collected; patients demographics (age, gender, BMI, and social status), date of a first symptomatic positive culture of UTI episode, presence of symptoms (dysuria, fever, flank pain, etc.), UTI risk factors (renal transplant, neurogenic bladder, and vesicoureteral reflux, etc.), antibiotic prophylaxis used (Nitrofurantoin, Augmentin, First-generation cephalosporin, Ampicillin, Amoxicillin, Fosfomycin, Trimethoprim, and Bactrim), antibiotic prophylaxis start and completion dates, a number of visits while on antibiotic prophylaxis, number of UTI while on antibiotic prophylaxis and in the follow-up period, and antibiotic prophylaxis compliance documentation.

## Data Analysis

The data was analyzed by Statistical Package for Social Sciences (SPSS software version 25). Categorical data (e.g., gender, and comorbidities) was presented by frequencies and percentages. Continuous variables such as age was presented by means and standard deviation. Descriptive and inferential statistics have been performed for the socio-demographic and clinical variables. The categorical variables were compared using a chi-square test or Fisher’s exact test, as appropriate. All tests were two-tailed, and significance was accepted at a *p*-value < 0.05.

## Results

**Fig. 1:**
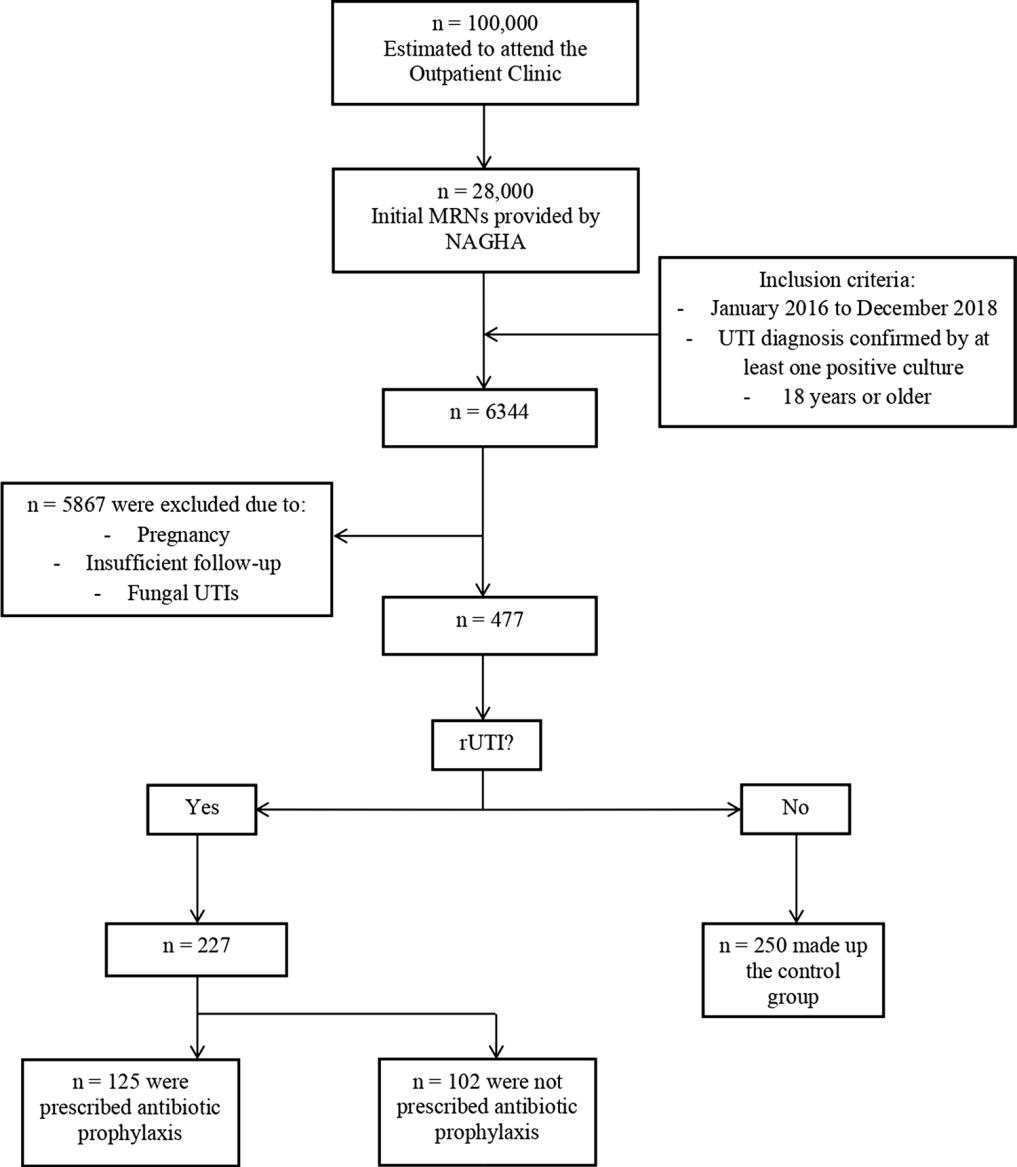
Process of subjects’ selection

A total of 477 patients with at least one symptomatic with a positive urine culture were seen in outpatient department (OPD) in the period between January 2016 and December 2018 (Fig. 1). There were 250 patients with a documented single UTI episode and 227 patients with recurrent UTI episodes during the study period (Table 1). The mean age of all patients with urinary tract infections was 57 ± 20.5 and with a mean body mass index (BMI) of 29.6 ± 10.3. Those with recurrent UTIs were significantly older with a mean age of 60.6 ± 21.54 vs 53.6 ± 19.1 (*p* < 0.001) (Table 1).

**Table 1:** Characteristics of patients in the recurrent and single urinary tract infections episodes

Parameter	Whole group *n* (%) *N* = 477	Single UTI *n* (%) *N* = 250	Recurrent UTI *n* (%) N = 227	*P-value*
Age	57 ± 20.5	53.6 ± 19.1	60.6 ± 21.54	< 0.001^b^
Age				
17–32	76 (16)	37 (48.7)	39 (51.3)	< 0.001
33–48	105 (22)	78 (74.3)	27 (25.7)	
49–64	103 (21.5)	51(49.5)	52 (50.5)	
≥ 65	193 (40.5)	84 (43.5)	109 (46.5)	
Gender				
Male	160 (33.5)	81 (50.6)	79 (49.4)	0.579
Female	317 (66.5)	169 (53.3)	148 (46.7)	
BMI (Mean ± SD)	29.6 ± 10.3	29.3 ± 7.1	30 ± 12.8	0.797
Social status				
Single	92 (19.5)	47 (51)	45 (49)	0.965
Married	347 (73)	186 (53.6)	161 (46.4)	
Divorced/Widowed	38 (7.5)	17 (44.7)	21 (55.3)	
First UTI pre menopause	136 (42.9)	92 (67.6)	44 (32.4)	< 0.001
First UTI post-menopause	131(41.3)	50 (38.2)	81 (61.8)	
No documentation	50 (15.7)	27 (54)	23 (46)	
Comorbidities				
DM	217 (45.5)	92 (42.4)	125 (57.6)	< 0.001
HTN	272 (57)	112 (41.2)	160 (58.8)	< 0.001
Chronic renal disease	122 (25.6)	29 (23.8)	93 (76.2)	< 0.001
Immunosuppressive drugs	108 (22.6)	36 (33.3)	72 (66.7)	< 0.001
Chronic steroid use	90 (18.8)	25 (27.8)	65 (42.2)	< 0.001
Heart failure	62 (13)	21 (34)	41 (66)	0.002
Mental health illness	46 (9.6)	12 (26)	34 (74)	< 0.001
Respiratory disease	67 (14.1)	31 (46.3)	36 (53.7)	0.278
Endocrine disease (non-diabetes)	111 (23.3)	49 (44)	62 (56)	0.046
Malignancy	71 (14.8)	43 (60.6)	28 (39.4)	0.136
Neurologic disease	57 (12)	25 (44)	32 (56)	0.168
Chronic liver disease	22 (4.6)	8 (36.4)	14 (63.6)	0.123
Chronic rheumatologic disease	19 (3.9)	8 (42)	11 (58)	0.359
Others	22 (4.8)	14 (63.6)	8 (36.4)	0.88
Risk factors				
History of urinary incontinence	52 (10.9)	14 (27)	38 (73)	< 0.001
Renal transplant	74 (15.5)	15 (20)	59 (80)	< 0.001
Recent indwelling urinary catheter	65 (13.6)	15 (23)	50 (77)	< 0.001
Intermittent catheterization	24 (4.8)	3 (12.5)	21 (87.5)	< 0.001
Chronic indwelling catheterization	43 (9)	10 (23.3)	33 (79.7)	< 0.001
Polycystic kidney disease	4 (0.8)	1 (25)	3 (75)	0.351^a^
Urological procedures	45 (9.4)	7 (15.7)	38 (84.4)	< 0.001
Immobilization	68 (14.3)	22 (32.4)	46 (67.6)	< 0.001
Neurogenic bladder	27 (5.6)	3 (11)	24 (89)	< 0.001
Organism isolated total				
*E. coli*	233 (48.8)	118 (50.6)	115 (49.4)	0.45
*Klebsiella pneumonia*	102 (21.4)	48 (47)	54 (53)	0.120
*Enterococcus faecalis*	44 (9.3)	28 (63.6)	16 (36.4)	0.118
*Pseudomonas aeruginosa*	17 (3.56)	6 (35.3)	11 (46.7)	0.216^a^
*Streptococcus agalactiae*	33 (6.9)	25 (75.7)	8 (24.3)	0.006^a^
Others	48 (10.1)	25 (52)	23 (48)	0.033
Urology referral	127 (26.6)	47 (37)	80 (63)	< 0.001
OB/GYN referral	104 (21.8)	60 (57.7)	44 (42.3)	0.232
Post void studies	9 (1.8)	1 (11.2)	8 (88.8)	0.016^a^
Topical estrogens use	8 (1.6)	0	8 (100)	NA
No. of prophylactic antibiotic courses				
No prophylaxis	102 (21.4)	–	102 (100)	NA
One course	105 (22)	–	105 (100)	
Two Course	20 (4.2)	–	20 (100)	
Antibiotic used prophylactic				
Nitrofurantoin	52 (10.9)	–	52 (100)	NA
Bactrim	65 (13.6)	–	65 (100)	

^a^*Fisher exact test*

^*b*^*Mann Whitney test*

More than two third of UTI patients were female, 317 (66.5%), of whom 62 (19.6%) are single. Recurrent UTI occurred in (*n* = 148, 46.7%) females while 169 (53.3%) had a single episode. Out of the 224 married females with urinary tract infections, (*n* = 125, 55.8%) had single episodes and (*n* = 99, 44.2%) had recurrent UTI (*P* = 0.75). First urinary tract infection occurred at pre-menopausal in 136 patients (42.9%) and in the post-menopause in 131 patients (41.3%). In patients with recurrent urinary tract infections, first episode of infections more frequently occurred at post-menopause (*P* < *0.001*) (Table 1).

The most frequently documented comorbidities in patients with urinary tract infections were hypertension, in 272 patients (57%), diabetes mellitus in 217 patients (45.5%) and chronic renal disease in 122 (25.6%) patients (Table 1). Comorbidities significantly associated with risk of recurrent urinary tract infections included, Diabetes, Hypertensions, Chronic renal disease, Use of immunosuppressive medications including steroids and biological drugs, heart failure and mental health illness (Table 1). Only Chronic renal disease and mental illness were associated with recurrent UTI in multivariate analysis (Table 2).

**Table 2:** Adjusted Odd ratio of risk factors of recurrent urinary tract infections

Variable	Total	*COR* (95% CI)	*P value*	*AOR* (95% CI)	*P value*
Age					
17–32	76 (16)	0.812 (0.48–1.38)	0.44	0.672 (0.27–1.69)	0.39
33–48	105 (22)	0.267 (0.158–0.45)	< 0.001	0.39 (0.18–0.86)	0.02
49–64	103 (21.5)	0.78 (0.48- 1.27)	0.325	0.57 (0.31–1.05)	0.07
≥ 65	193 (40.5)	1		1	
Comorbidities					
DM	217 (45.5)	2.11 (1.46–3.04)	< 0.001	1.15 (0.62–2.16)	0.65
HTN	272 (57)	2.94 (2.02–4.29)	< 0.001	1.41 (0.73–2.68)	0.31
Chronic renal disease	122 (25.6)	5.29 (3.31–8.45)	< 0.001	2.30 (1.19–0.46)	0.01
Immunosuppressive drugs	108 (22.6)	2.76 (1.76–4.33)	< 0.001	1.67 (0.72–3.89)	0.23
Chronic steroid use	90 (18.8)	3.61 (2.18–5.97)	< 0.001	0.91 (0.37–2.23)	0.84
CHF	62 (13)	2.41 (1.37–4.2)	0.002	1.64 (0.81–3.33)	0.17
Mental health illness	46 (9.6)	3.49 (1.76–6.93)	< 0.001	2.76 (1.18–6.16)	0.02
Respiratory disease	67 (14.1)	1.33 (0.79–2.24)	0.28	1.24 (0.65–2.34)	0.52
Endocrine disease (non-diabetes)	111 (23.3)	1.54 (1.01–2.36)	0.05	1.55 (0.87–2.74)	0.14
Malignancy	71 (14.8)	0.67 (0.41–1.13)	0.14	0.69 (0.34–1.44)	0.33
Neurologic disease	57 (12)	1.47 (0.85–2.57)	0.17	0.81 (0.38–1.73)	0.58
Chronic liver disease	22 (4.6)	1.98 (0.82–4.83)	0.13	1.76 (0.57–5.45)	0.33
Chronic rheumatologic disease	19 (3.9)	1.54 (0.61–3.9)	0.36	1.65 (0.52–5.3)	0.39
Risk factors					
History of urinary incontinence	52 (10.9)	3.39 (1.78–6.4)	< 0.001	2.86 (1.3–6.29)	0.009
Renal transplant	74 (15.5)	5.5 (3.02–10.03)	< 0.001	3.32 (1.31–8.44)	0.012
Recent indwelling urinary catheter	65 (13.6)	4.43 (2.41–8.14)	< 0.001	4.05 (1.49–11.02)	0.006
Intermittent catheterization	24 (4.8)	8.39 (2.47–28.54)	0.001	5.52 (1.29–23.6)	0.021
Chronic indwelling catheterization	43 (9)	4.08 (1.96–8.49)	< 0.001	1.36 (0.43–4.29)	0.59
Polycystic kidney disease	4 (0.8)	3.34 (0.34–32.29)	0.29	2.38 (0.15–39.1)	0.54
Urological procedures	45 (9.4)	6.98 (3.05–15.97)	< 0.001	2.9 (1.04–8.12)	0.04
Immobilization	68 (14.3)	2.6 (1.53–4.54)	< 0.001	2.39 (1.18–4.84)	0.02
Neurogenic bladder	27 (5.6)	9.73 (2.89–32.79)	< 0.001	4.39 (0.95–20.15)	0.02

Risk factors of urinary tract infections that poses increase risk of recurrent infections included history of urinary incontinence, renal transplantation, recent indwelling or intermittent urinary catheterization, urological procedures, immobilization and neurogenic bladder were all associated with risk of recurrent urinary tract infection (Table 2).

The most common uropathogens isolated in urinary culture were E. coli, 233 patients (49%), Klebsiella pneumoniae, 102 patients (21%), and Enterococcus faecalis, 44 patients (9.2%) (Table 1). E. coli infections was the most prevalent organism in both patients who have single episode and recurrent UTI episodes. Streptococcus agalactiae was more significantly associated with single UTI episode (*p* = 0.006).

At least one course of continuous prophylactic antibiotics was prescribed in 125 patients, 55 (44%) of those were post-renal transplant patients. In the post-renal transplant group, 17 patients (31%) were given prophylactic antibiotic after a single episode of UTI and 39 patients (71%) were given continuous prophylaxis after multiple UTI episodes. There were 10 other patients who received continuous antibiotic prophylaxis course after a single episode of UTI, mainly in patients with chronic urinary tract catheterization, neurogenic bladder and vesicoureteral reflux disease.

Antibiotic prophylaxis was not given in 102 (45%) patients with recurrent UTI (Table 3). The mean age of those not receiving prophylactic was significantly older, 67.2 ± 20.1 vs 55 ± 21 (*p* < 0.001). Patients above age of 65 were less likely to receive prophylaxis (*p* < 0.001). Renal transplantation, history of urological procedures, intermittent urinary catheterization and neurogenic bladder were all significantly associated with decision to use continuous prophylaxis antibiotics while immobilization was significantly associated with avoiding the use of prophylaxis therapy (Table 3). The mean number of UTI episodes in patients who did not receive antibiotics prophylaxis was (4.7 ± 3.5) episodes vs (3.3 ± 3.1) episodes in those who received antibiotics (*P* < *0.001*).

**Table 3:** Comparison of patients with recurrent UTI with or without continuous prophylaxis antibiotics

Parameter	DID NOT receive prophylactic antibiotics *n* (%) *n* = 102	Received prophylaxis Antibiotics *n* (%) *n* = 125	*P value*
Age (Mean ± SD)	67 ± 20	55 ± 21	< 0.001
Age			
17–32	8 (7.8)	31(24.8)	< 0.001
33–48	11 (10.8)	16 (12.8)	
49–64	19 (18.6)	33(26.4)	
≥ 65	64 (62.7)	45 (36)	
Gender			
Male	40 (39.2)	39 (31.2)	0.207
Female	62 (60.8)	86(68.8)	
Risk factors			
History of urinary incontinence	19 (18.6)	19 (15.2)	0.491
Renal transplant	4 (3.9)	55 (44)	** < 0.001**
Recent indwelling urinary catheter	24 (23.5)	26 (20.8)	0.622
Intermittent catheterization	4 (3.9)	17 (13.6)	**0.012**
Chronic indwelling catheterization	19 (18.6)	14 (11.2)	0.114
Polycystic kidney disease	1 (1)	2 (1.6)	0.684^a^
Urological procedures	5 (4.9)	33 (26.4)	** < 0.001**
Immobilization	29 (28.4)	17 (13.6)	**0.008**
Neurogenic bladder	3 (2.9)	21 (16.8)	**0.001**
Comorbidities			
DM	58 (56.9)	67 (53.6)	0.623
HTN	73 (71.6)	87 (69.6)	0.746
Chronic renal disease	24 (23.5)	69 (55.2)	< 0.001
Immunosuppressive drugs	15(14.7)	57 (45.6)	< 0.001
Chronic steroid use	10 (9.8)	55 (44)	< 0.001
CHF	19 (18.6)	22 (17.6)	0.841
Mental health illness	10 (9.8)	24 (19.2)	0.048
Respiratory disease	18 (17.6)	18 (14.4)	0.505
Endocrine disease (non-diabetes)	20 (19.6)	42(33.6)	0.019
Malignancy	18 (17.6)	10 (8)	0.028
Neurologic disease	15 (14.7)	17 (13.6)	0.85
Chronic liver disease	5 (4.9)	9 (7.2)	0.474
Chronic rheumatologic disease	3 (2.9)	8 (6.4)	0.353
Others	7 (6.9)	5 (4)	0.141
First UTI pre menopause	16(15.7)	28 (22.4)	0.037
First UTI post-menopause	42 (41.2)	39 (31.2)	
No documentation	5 (4.9)	18 (14.4)	
Organism isolated total			
E. coli	49 (48)	65 (52.4)	0.475
Klebsiella pneumoniae	30 (29.4)	24 (19.4)	0.072
Enterococcus faecalis	7 (6.9)	12 (9.7)	0.609^a^
Pseudomonas aeruginosa	6 (5.9)	5 (4)	0.548^a^
Streptococcus agalactiae	3 (2.9)	5 (4)	0.732^a^
Urology referral	24 (23.5)	56 (44.8)	0.001
OB/GYN referral	14 (13.7)	30 (24.2)	0.056
Post void studies	0	8 (6.5)	NA
Topical estrogens use	2 (2)	6 (4.8)	0.301
Number of UTI episodes (Mean ± SD)	4.7 ± 3.5	3.4 ± 3.1	0.005

^*a*^*Fisher exact test*

^*b*^*Mann Whitney test*

The most frequently used antibiotic prophylaxis for those prescribed a prophylaxis antibiotic was oral trimethoprim/sulfamethoxazole (TMP-SMX), prescribed for 65 patients (52%) followed by oral Nitrofurantoin, prescribed for 52 patients (41.6%). Amoxicillin/clavulanic acid was prescribed in eight patients only (6.4%). The mean duration of antibiotics prophylaxis was (128 ± 58) days for Bactrim, (93 ± 46) days for Nitrofurantoin and (58 ± 42) days for Amoxicillin/clavulanic acid. The most frequent first prescribers for prophylaxis antibiotics were nephrologist followed by urologist and infectious disease specialist. Prophylactic antibiotics were prescribed more in renal transplant patients (*P* < 0.001), neurogenic bladder patients (*P* < *0.001*) and those with urological pathology (*P* < 0.001).

TMP-SMX was more prescribed in younger patients, (49 ± 18.8 vs 63.4 ± 21.5), *P* < 0.001), in post- renal transplantation (*P* < *0.001*) and after urological procedures (*P* < *0.001*), while Nitrofurantoin was more prescribed in immobilized patients (*P* = 0.002) and in patients with neurogenic bladder (*P* < *0.001*) (Table 4). TMP-SMX was prescribed significantly more for recurrent UTI occurring in the pre-menopausal period.

**Table 4:** Factors contributing to the use of Nitrofurantoin vs Bactrim as the antibiotic regimen for prophylaxis

Parameter	*Antibiotic A* Nitrofurantoin *N* = 52	*Antibiotic B* Bactrim *N* = 65	*P-value*
Age			
17–32	8 (15.4)	20 (30.7)	** < 0.001**
33–48	4 (7.6)	11 (16.9)	
49–64	13 (25)	19 (29.2)	
≥ 65	27 (52)	15 (23.1)	
Age (Mean ± SD)	63.4 ± 21.5	49 ± 18.8	** < 0.001**
Gender			
Male	16 (30.7)	21 (32.3)	0.622
Female	36 (69.3)	44 (67.7)	
Risk factors			
History of urinary incontinence	13 (25)	6 (9.6)	0.073
Renal transplant	7 (13.5)	42 (64.6)	< 0.001
Recent indwelling urinary catheter	11 (21.2)	15 (23)	0.956
Intermittent catheterization	9 (17.3)	8 (12.3)	0.012^a^
Chronic indwelling catheterization	9 (17.3)	4 (6.2)	0.059^a^
Polycystic kidney disease	1 (1.9)	2 (3.1)	1^a^
Urological procedures	2 (3.8)	30 (46.2)	< 0.001
Immobilization	13 (25)	3 (4.6)	0.002
Neurogenic bladder	15 (28.8)	6 (9.2)	< 0.001

^*a*^*Fisher exact test*

^*b*^*Kruskal-Wallis*

Patients who received continuous prophylactic antibiotics experienced significantly less symptomatic episodes of urinary tract infections (*P* < 0.001), Emergency room visits and hospital admissions due to urinary tract infections (*P* < *0.001*) for both Nitrofurantoin and TMX-SMX. (Tables 5, 6).

**Table 5:** Frequency of UTI episodes prior and post antibiotic prophylaxis therapy in the period of 12 months

Number of UTI episodes	Number of patients pre-therapy *n* (%)	Number of patients after completion of treatment *n* (%)	*P-value*
*Antibiotic A* Nitrofurantoin N = 52			
One	6 (11.5)	4 (7.7)	< 0.001*
Two	8 (15.4)	4 (7.7)	
Three	10 (19.2)	3 (5.7)	
Four	4 (7.7)	3 (5.7)	
≥ Five	24 (46)	9 (17.3)	
*Antibiotic B* Bactrim N = 65			
One	23 (35.4)	12 (18.4)	< 0.001*
Two	14 (21.5)	6 (9.2)	
Three	5 (7.7)	4 (6.1	
Four	5 (7.7)	3 (4.6)	
≥ Five	9 (13.8)	4 (6.1)

**Table 6:** Emergency room visits and hospital admissions due to UTI pre and post prophylactic antibiotics

No. ER visit	Number of patients pre-therapy *n* (%)	Number of patients after completion of treatment *n* (%)	*P value*	No. hospital admissions	Number of patients pre-therapy *n* (%)	Number of patients after completion of treatment *n* (%)	*P-value*
*Antibiotic A* Nitrofurantoin *N* = 52							
None	17 (32.7)	35 (67.3)	0.001*	None	21 (40.4)	37 (71.2)	0.001*
One	13 (25)	8 (15.4)		One	14 (27)	9 (17.3)	
Two	12 (23)	6 (11.5)		Two	8 (15.4)	5 (9.6)	
Three	6 (11.5)	3 (5.8)		Three	5 (9.6)	1 (1.9)	
≥ Four	4 (3.8)	0		≥ Four	4 (3.8)	0	
*Antibiotic B* Bactrim *N* = 65							
None	29 (44.6)	47 (72.3)	< 0.001*	None	30 (46.2)	50 (76.9)	< 0.001*
One	15 (23)	10 (15.4)		One	17 (26.2)	10 (15.4)	
Two	10 (15.4)	5 (7.7)		Two	8 (12.3)	3 (4.6)	
Three	6 (9.2)	2(3.1)		Three	6 (9.2)	2 (3.1)	
≥ Four	5 (4.6)	1 (1.5)		≥ Four	4 (3.1)	0	

There was no documentation on any education given to patients with UTI or recurrent UTI in regard to how to avoid recurrent infections. Referral for Gynecological evaluation was requested for 44 female patients (20%) with recurrent UTI, of whom 11 (25%) and 27 (61%) were pre-menopausal and postmenopausal, respectively.

Urology referral was requested in 80 female patients with recurrent UTI (35.2%); 19 patients (23.7%) and 20 patients (25%) were pre-menopausal and postmenopausal, respectively.

Urological evaluation was requested for 36 male patients (45.6%) with recurrent UTI (45.6%). Male gender with recurrent urinary tract infections were more frequently referred to Urology clinic compared to female gender (*P* = 0.042).

## Discussion

This study explores a very complex and common problem in medical practice in Saudi Arabia, which is recurrent urinary tract infection. One relevant previous study in adults explored host-related risk factors for recurrent urinary tract infection in Saudi women of childbearing age but did not elaborate on management strategies used [15]. We described the risk factors and the use of antibiotic prophylaxis in patients with recurrent UTI in Saudi Arabia. We showed that old age, female gender and comorbidities (diabetes, indwelling urinary catheterization, immobilization, neurogenic bladder and renal transplantation) were associated with recurrent urinary tract infection. The use of antibiotic prophylaxis was associated with a reduction in the number of episodes of urinary tract infection and a decrease in the emergency room visits and hospitalization for UTI. The identified risk factors for recurrent UTI in our study were similar to previous studies nationally and internationally [15, 22–25].

Being conducted in a tertiary care center, almost half of patients who received antibiotic prophylaxis for recurrent UTI were renal transplants, in addition to patients with neurogenic bladder, bladder flow obstruction and intermittent catheterization. This population sitting may explain the higher percentage of male patients with recurrent urinary tract infections in our study compared to other studies conducted in the primary care setting [22, 25–27]. Our results are useful to improve the care of patients with recurrent UTI in tertiary care centers being common and affecting patients with different comorbidities but may not be applicable for pre-menopausal healthy female patients in the primary care setting to. The efficacy of antibiotic prophylaxis in patients with recurrent UTI is conflicting in various studies while some studies showed more harm than benefit is noted in view of development of resistant pathogens and Clostridium Difficile associated diarrhea, other studies showed efficacy in preventing recurrent urinary tract infections in patients with intermittent catheterization [28–30].

Only half of patients with recurrent UTI were prescribed prophylaxis antibiotics. These patients were transplant recipients, with neurogenic bladder and intermittent catheterization. Although we showed a reduction of recurrent UTI in patients who received antibiotic prophylaxis, our sample size was not powered to assess the group of patients that would benefit significantly from antibiotic prophylaxis. Both TMP-SMX and Nitrofurantoin were used effectively to prevent recurrent UTI in our study. It is not clear whether the practice of choosing one antibiotic over the other was based on patient characteristics, pathogen resistance pattern or physician versus patient preference. Fosfomycin was not used in our study in view of unavailability. Future studies are needed to evaluate the practice of various antibiotic options used as prophylaxis for recurrent UTI with emphasis on emerging bacterial resistance.

We noted that only one third of patients with recurrent UTI were referred for urological and gynecological evaluation in our study. In addition, the use of vaginal estrogen cream in postmenopausal female patients with recurrent UTI is limited. The concept of using antibiotic prophylaxis in preventing recurrent UTI without urological and gynecological review in our study is an indicator for the urgent need to develop a multi-disciplinary care for patients with recurrent UTI especially in the era of increasing bacterial resistance in tertiary care centers.

Patients with recurrent urinary tract infection in our cohort were significantly older more than those with single episode which is consistent with other report from Saudi showing recurrence UTI more frequent in those above age of 65 years [15] and international literature showing increasing prevalence of 20% with in women who are 65 years and older [15, 21, 31].

Although recurrent UTI is more frequent among females occurring in 65.2% of our sample population, more than one third (34%) of recurrent urinary tract infections episodes in this patient’s population occurred in males. This is higher than what is found by Ahmad eta al who reported retrospectively in 19,696 adults in that 20% of adults with recurrent urinary tract infections were male [31]. Our patient’s population are from a tertiary care hospital outpatient clinic compared to a community set up in the previous reference and a much smaller number in our study, all of which may explain the prevalence difference in male patients. Recurrent urinary tract infections are not infrequent in male patients however; but still occur far less than female patients. Around 14% of men will experience at least one UTI in their lifetime, compared with approximately 50% of women. Prevalence of urinary tract infection increase in male above age of 85 and 15% of those with UTI will have recurrent infection. Recurrent urinary tract infections in male are more likely related to prostatic hypertrophy and diabetes both of which lead to high post-void residuals [32, 33] The mean age of our male patients with recurrent UTI was (64.6 ± 22.5) years.

Risk factors for recurrent urinary tract infection included diabetes, indwelling urinary catheterization, immobilization, neurogenic bladder and renal transplantation and are all consistent with what is previously reported [22, 23, 25, 34]. Because of increased risk of recurrent UTI in post-transplant especially in the first few months’ post-transplant [25], many physicians elected to start prophylaxis therapy even before the first episode or after a single episode of urinary tract infection in this population. Among all the risk factors, we found that renal transplant patients had a high rate of antibiotic prophylaxis prescription despite having a single UTI episode in our cohort.

Over all Trimethoprim-Sulfamethoxazole was the most frequently used prophylaxis antibiotic in both men and women although Nitrofurantoin is still used more frequently in our patients compared to other studies specially in male (18% vs 31%) [31]. TMP-SMX is also the most prescribed antibiotic prophylaxis in post-renal transplant adult patients compared to Nitrofurantoin (*P* = *0.019*). At the time of this study, Fosfomycin was not present in our formulary list which can explains the low use of this drug.

Both Nitrofurantoin and TMP-SMX were effective in reducing number of symptomatic urinary tract infections, admissions to hospital and ER visits due to UTI in our patients. Similar results were found by Philipp *Jent *et al. who conducted a systematic review and meta-analysis of published randomized controlled trials and concluded that continuous prophylactic antibiotics are effective in reducing recurrent infections irrespective of the antibiotics used [35]. Continuous antibiotic prophylaxis was also is effective in reducing recurrent UTI in patients who use clean intermittent self-catheterization [36]. Antibiotics regimens used included Nitrofurantoin, trimethoprim and Bactrim resulted in close to 50% reduction in the rate of UTI episodes.

The benefit continuous prophylaxis antibiotics in elderly patients was studied previously with conflicting results. Some studies showed increased risk of harm due to resistant organisms and Clostridioides difficile infection [37] while antibiotic prophylaxis was associated with an average of 50% reduction in UTI in men and women above age of 65 in others [31]. In this study, there were 42 patients above age of 65 years who received continuous prophylaxis. Nitrofurantoin was more frequently used in this patient population (64.3% vs 35.7%).

Our results show a low prescription of topical estrogen as a preventative measure for recurrent UTI in postmenopausal women. Out of 227 patients with recurrent UTI only 7 patients (3.1%) were prescribed topical estrogen. The lack of referrals to the gynecological department in our data correlates with the low prescription of topical estrogen. This contraindicates with recent studies proving that topical estrogen is an effective conservative treatment of recurrent UTI in postmenopausal women [38, 39].

Chang et al. prioritized topical estrogen as a first line treatment for postmenopausal women with recurrent UTI [39].

Measures other than antibiotic prophylaxis have been proposed to be just as effective as prophylactic antibiotics. Harding et al. proposed that Methenamine Hippurate is not inferior to antibiotic prophylaxis in terms of UTI prevention as it showed comparable efficacy, as well as it negates the risk of antimicrobial resistance [40]. Those measures were rarely used or documented as part of alternatives for our patients.

The main limitation of our study is being single center and retrospective in nature with selection bias. In addition, the lack of antibiotic susceptibility patterns of bacterial isolates of UTI is a major limitation of our study. Our main strength is the detailed evaluation of risk factors of recurrent UTI in relation to the use of antibiotic prophylaxis.

## Conclusion

Urinary tract infection and recurrent UTI are common problems in outpatient’s visits especially in postmenopausal women. About one third of patients with recurrent UTI are male. Continuous antibiotic prophylaxis was only used in about half of recurrent UTI patients with TMP-SMX being the most frequently used. It was effective in reducing recurrence rate, ER visits and hospital admissions due to recurrent UTI. Urology and gynecological referral were infrequently requested as part of the evaluation process for patients with recurrent UTI. There was a lack of use of other interventions such as topical estrogen in postmenopausal women. The overall process of caring of patients with recurrent UTI lacks adequate documentations and focus on individuals’ preference rather than an organized systematic approach.

## Data Availability

Not available.

## Abbreviations

UTI: Urinary tract infection
OP: Odds ratio
RCTs: Randomized clinical trials
NGHA: National Guard-Health affairs
OPD: Outpatient clinic
BMI: Body mass index
SPSS: Statistical Package for Social Sciences
TMP-SMX: Trimethoprim/sulfamethoxazole
ER: Emergency room ER
